# In vitro antioxidant and antimalarial activities of leaves, pods and bark extracts of *Acacia nilotica* (L.) Del.

**DOI:** 10.1186/s12906-017-1878-x

**Published:** 2017-07-18

**Authors:** Muhammad Bilal Sadiq, Pattamon Tharaphan, Kesinee Chotivanich, Joel Tarning, Anil Kumar Anal

**Affiliations:** 1 Food Engineering and Bioprocess Technology, Asian Institute of Technology, Klongluang, PO Box 4, Bangkok, Pathumthani 12120 Thailand; 2 Mahidol-Oxford Tropical Medicine Research Unit, Faculty of Tropical Medicine, Mahidol University, Bangkok, Thailand; 3 Department of Clinical Tropical Medicine, Faculty of Tropical Medicine, Mahidol University, Bangkok, Thailand; 4 Centre for Tropical Medicine and Global Health, Nuffield Department of Clinical Medicine, University of Oxford, Oxford, UK

**Keywords:** *Acacia nilotica*, *Plasmodium falciparum*, Malaria, Antioxidants, Mature schizonts

## Abstract

**Background:**

The emergence of drug resistant malaria is threatening our ability to treat and control malaria in the Southeast Asian region. There is an urgent need to develop novel and chemically diverse antimalarial drugs. This study aimed at evaluating the antimalarial and antioxidant potentials of *Acacia nilotica* plant extracts**.**

**Methods:**

The antioxidant activities of leaves, pods and bark extracts were determined by standard antioxidant assays; reducing power capacity, % lipid peroxidation inhibition and ferric reducing antioxidant power assay. The antimalarial activities of plant extracts against *Plasmodium falciparum* parasites were determined by the 48 h schizont maturation inhibition assay. Further confirmation of schizonticide activity of extracts was made by extending the incubation period up to 96 h after removing the plant extract residues from parasites culture. Inhibition assays were analyzed by dose-response modelling.

**Results:**

In all antioxidant assays, leaves of *A. nilotica* showed higher antioxidant activity than pods and bark. Antimalarial IC_50_ values of leaves, pods and bark extracts were 1.29, 4.16 and 4.28 μg/ml respectively, in the 48 h maturation assay. The IC_50_ values determined for leaves, pods and bark extracts were 3.72, 5.41 and 5.32 μg/ml respectively, after 96 h of incubation. All extracts inhibited the development of mature schizont, indicating schizonticide activity against *P. falciparum*.

**Conclusion:**

*A. nilotica* extracts showed promising antimalarial and antioxidant effects. However, further investigation is needed to isolate and identify the active components responsible for the antimalarial and antioxidant effects.

## Background

*Acacia nilotica* (L.) Del. is a medicinal plant belonging to the family Fabaceae. The plant is widely distributed in tropical and subtropical regions. *A. nilotica* is rich in bioactive compounds and used for prevention and treatment of various ailments and infectious diseases. In ayurvedic medicine practice, it is believed that leaves, bark and pods of *A. nilotica* can be used against cancer, diarrhea, fever and menstrual problems [1]. The plant is rich in polyphenolic compounds, in which catechins are hypothesized to possess antioxidant and anti-inflammatory activities [2]. *A. nilotica* has been reported to have inhibitory effect against hepatitis C virus protease [3] and multidrug resistant bacterial pathogens [4]. In aerial parts of the plant, a variety of phenolic compounds were identified with a wide range of biological activities [5]. In recent years, researchers have tried to isolate strong, nontoxic antioxidants from edible plants to prevent autoxidation and lipid peroxidation with the aim to replace synthetic antioxidants [6]. Plant extracts containing high amounts of bioactive compounds especially antioxidants, have the potential of being used in food, agriculture, nutraceuticals, cosmetics and pharmaceutical products [7].

Malaria is a major parasitic disease, accountable for approximately 214 million new clinical cases and 0.44 million deaths, globally in 2015. The majority of deaths occur in children under the age of five [8]. Most of the cases were reported in Sub-Saharan Africa (88%), followed by Southeast Asia (10%) and the Eastern Mediterranean region (2%).

The introduction of artemisinin-based combination therapy has resulted in a substantial decline in malaria during the last decade. Artemisinin, originally derived from *Artemisia annua*, is the most potent antimalarial class of drugs available today, and they exhibit a substantially faster elimination of parasites compared to other compounds [9]. However, the recent gains in antimalarial therapy are threatened by the emerging artemisinin-resistant *P. falciparum* malaria in Southeast Asia. Consequently, new drugs and drug combinations are urgently needed for the treatment of malaria. Ideally, these drugs should have novel modes of action and be chemically different from drugs in current use [10]. The use of medicinal plants against various infectious diseases is an age-long practice, and could provide novel compounds and drug classes.

The aim of the present work was to investigate the antioxidant and antimalarial properties of *A. nilotica*, traditionally used as medicinal plant.

## Methods

### Preparation of plant extracts

Leaves, pods and bark of the *A. nilotica* plant (wild) were collected from Lahore, Pakistan. The plant was authenticated by Associate Prof. Dr. Hamad Ashraf and the voucher specimen (Voucher number S6 HbGCS with Reference number 7998) was deposited to Botany Society Government College of Science, Lahore, Pakistan. The plant samples were washed thoroughly with sterilized distilled water and dried under shade for 48 h. The extraction of plant samples was conducted following the method as described by Adwan et al. [11] with slight modifications. Powdered plant samples (25 g) were placed in 250 ml of ethanol (80%, *v*/v) in conical flasks and placed on shaking incubator (Gallenkamp, UK) at 200 rpm for 72 h at 25 °C. The extracts were filtered and concentrated by means of a rotary evaporator (Büchi rotavapor R-144, Switzerland) followed by lyophilization for 24 h in a freeze dryer (Scanvac Cool Safe 55–4, Denmark). The freeze dried extracts were stored at 4 °C until further use. The stock solutions (2 mg/ml) of extracts were freshly prepared in ethanol (40%, *v*/v) prior to each experiment.

### Antioxidant activity of *A. nilotica* extracts

#### Determination of reducing power capacity of plant extracts

The reducing power capacity of *A. nilotica* was estimated by the method as described by Nabavi et al. [12], with slight modifications. Increasing concentrations (125 to 1000 μg/ml) of each extract were prepared from a stock solution. The plant extract (2.5 ml) was mixed with phosphate buffer solution (PBS; 0.2 M, pH 6.6, 2.5 ml) and potassium ferricyanide (2.5 ml, 1% *w*/*v*), and incubated at 50 °C for 20 min. The reaction was stopped by adding trichloro acetic acid (TCA; 2.5 mL, 10% *w*/*v*) to the reaction mixture. The reaction mixture was centrifuged at 940×g for 10 min and the supernatant (2.5 ml) was mixed with ultra-purified water (2.5 ml) and ferric chloride (0.5 ml, 0.1%, *w*/*v*) solution. The absorbance was then measured at 700 nm by UV- visible spectrophotometer (UNICAM UV/Vis Spectrophotometer, UK), using ascorbic acid as positive control.

#### Determination of lipid peroxidation capacity of plant extracts

Lipid peroxidation activity of *A. nilotica* extracts was evaluated by thiobarbituric acid reactive substances (TBARS) assay following the method of Ohkawa et al. [13] with slight modifications. The egg yolk homogenate (10% *v*/v, in ultrapure distilled water) was used as lipid rich media. The different concentrations (125–2000 μg/ml) of *A. nilotica* extracts were prepared from stock solution. The plant extract (50 μl) was mixed with egg homogenate (250 μl) in test tube and volume was adjusted up to 500 μl by distilled water. Finally ferrous sulfate (25 μl, 0.07 M) was added to reaction mixture and incubated at 25 °C for 30 min to induce lipid peroxidation. Following the incubation period, 750 μl of acetic acid (20% *v*/v, pH 3.5), 750 μl of thiobarbituric acid (0.8% *w*/*v* prepared in 1.1% *w*/*v* sodium dodecyl sulfate) and 25 μl of trichloro acetic acid (20% *w*/*v*) were added to reaction mixture and incubated further for 60 min in boiling water bath. After cooling, 1-butanol (3 ml) was added and subjected to centrifugation at 940×g for 10 min. The upper organic layer was used to measure the absorbance at 532 nm by UV- visible spectrophotometer. Lipid peroxidation inhibition activity was evaluated by using the non-linear regression functionality in GraphPad Prism (dose-response analysis) [14].

#### Determination of ferric reducing antioxidant power capacity of plant extracts

Ferric reducing antioxidant power (FRAP) assay was done by the method given by Wong et al. [15], with slight modifications. FRAP reagent was prepared by mixing acetate buffer (pH 3.6), 2, 4, 6-tripyridyl-s-triazine (TPTZ, 10 mM) solution prepared in hydrochloric acid (40 mM) and ferric chloride solution (20 mM) in proportions of 10:1:1 (*v*/v), respectively. The FRAP reagent was prepared freshly every time and warmed at 37 °C prior to use. The plant extract (50 μl of 1 mg/ml) was added to FRAP reagent (1.5 ml) and incubated for 4 min at 37 °C. Absorbance was measured at 593 nm by UV- visible spectrophotometer and ferrous sulfate solution (100–1000 μM) was used to develop a standard curve. Results were expressed as μM of Fe (II)/g of dried extract. All experiments were conducted in triplicates and vitamin C was used as positive control.

### Antimalarial activity of *A. nilotica* extracts

Activity of plant extracts against *P. falciparum* (3D7; susceptible to artesunate) was evaluated by the schizont maturation inhibition assay (WHO standard test) following the method described by Chotivanich et al. [16] with slight modifications. The stock solutions of plant extracts were filtered through 0.45 μm micro filters and diluted in malarial culture medium (RPMI-1640 supplemented with 0.5% Albumax) to achieve 320 μg/ml. A stock solution of artesunate (60 mg/ml) was similarly diluted in RPMI-1640 to achieve a concentration of 320 ng/ml. Plant extracts (320 μg/ml) and artesunate (320 ng/ml) were then subjected to two-fold serial dilutions to obtain final concentrations, ranging from 160 to 0.156 μg/ml for plant extracts and 160 to 0.156 ng/ml for artesunate in 96 wells drug plates (three drug plates were used, each plate containing three replicates of each extract and three replicates of artesunate), with the first row of the plate kept as negative control (no drug). *P. falciparum* was cultivated in erythrocytes as host cells in RPMI-1640 supplemented with 0.5% Albumax. *P. falciparum* was maintained in the ring stage as the main experimental stage and synchronized with 5% sorbitol. A 75 μl cell suspension (10% hematocrit and 1% parasitemia) was introduced in each drug pre-coated and controlled well and incubated at 37 °C in the presence of 5% CO_2_ for 48 h. Following the incubation period cells were harvested and stained by Field’s stain method. The number of schizonts (≥8 merozoites/schizont) was counted per 100 infected cells. After harvesting at 48 h, cells were washed three times with buffer to eliminate any residual drug/extract and re-incubated with fresh malarial culture medium for further 48 h to observe the parasite growth. Antimalarial activity was evaluated by using the non-linear regression functionality in GraphPad Prism (dose-response analysis).

### Statistical analysis

All experiments were carried out in triplicates and results are expressed as mean values with standard deviation (± SD) of three replicates. One-way analysis of variance (ANOVA) and Tukey tests were carried out to determine significant group differences (*p* < 0.05) between means by using SPSS statistical software package (SPSS, version 16.0). Lipid peroxidation and antimalarial activities were evaluated by using the non-linear regression functionality in GraphPad Prism® version 6.01 (San Diego, US), i.e. dose vs. normalized response model with a variable slope. IC_50_ and Hill-slope values were computed by fitting relative activity at different concentrations of plant extracts and controls. The IC_50_ value was defined as concentration of tested drug or plant extract resulting in 50% of maximum effect compared to negative control (no drug). The Hill-slope was defined as the steepness of the dose-response curve.

## Results and discussion

### Antioxidant activity of *A. nilotica* extracts

#### Reducing power capacity of plant extracts

**Fig. 1:**
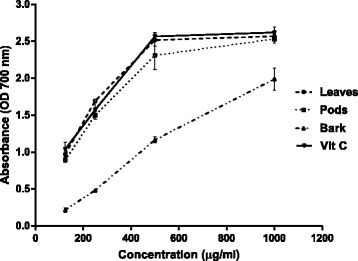
Reducing capacity of leaves, pods and bark extracts of *A. nilotica*

There was considerable variation in the reducing potential among the various parts of *A. nilotica* (Fig. 1). The reduction of ferric (Fe^+3^) to ferrous (Fe^+2^) in ferricyanide complex by the presence of antioxidants resulted in the color change of test solution from yellow to blue. This change in color of reaction mixture indicates the reducing potential of extracts. Moreover, reducing power capacity of extracts increased in a concentration-dependent manner. The reducing power capacities of leaves, pods and bark extracts were 2.57 ± 0.03, 2.53 ± 0.06 and 1.99 ± 0.15, respectively, and were not significantly different (*p* < 0.05) from the ascorbic acid at 1000 μg/ml. The positive control, ascorbic acid showed almost similar reducing capacity (2.62 ± 0.07) at 1000 μg/ml.

Addition of plant extracts to test solution during observing the reducing power assay, resulted in color change from yellow to green and blue. This shift in color indicated a reducing power capacity of plant extracts. The reducing compounds in extracts showed an antioxidant activity by donating hydrogen atoms and thus blocking the free radical chain reaction [17]. Kalaivani and Mathew [18], reported that ethanol fractions of *A. nilotica* leaves extract showed a reducing potential equivalent to the positive control (Vitamin C) at concentration of 100 μg/ml.

#### Lipid peroxidation capacity of plant extracts

**Fig. 2:**
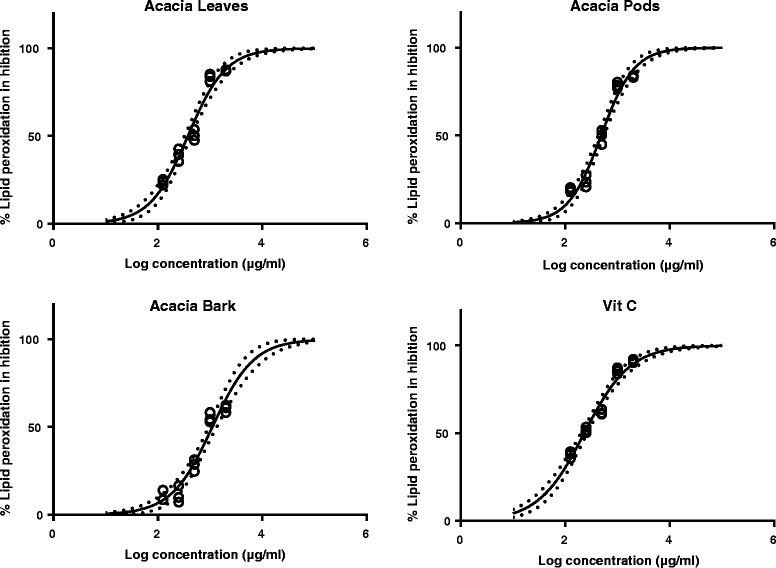
Estimated lipid peroxidation inhibition of *Acacia nilotica* extracts (leaves, pods and bark) and ascorbic acid (standard) at different concentrations, using non linear regression. Open circles represent observations, the solid lines represent the estimated mean curves, and the broken lines represent the 95% confidence intervals of the mean estimates

All extracts of acacia inhibited lipid peroxidation, induced by ferrous sulfate in egg yolk homogenate in a concentration-dependent manner. There was a considerable variation in lipid peroxidation inhibition among the various parts of acacia (Fig. 2). The highest inhibition (87.39 ± 0.41%) was found by leaves extract at 2000 μg/ml, whereas it was 83.72 ± 0.61% and 60.53 ± 2.05% for pods and bark, respectively. The positive control, L-ascorbic acid, showed comparatively higher inhibition of lipid oxidation (91.25 ± 0.97%) at 2000 μg/ml as compared to different parts of acacia.

A lower IC_50_ value represents a higher potential for inhibition of lipid peroxidation (Table 1). The leaves extract showed the lowest IC_50_ value of 375.5 (326.6–431.8) μg/ml, whereas, pods and bark extracts showed IC_50_ values of 486.5 (431.5–548.6) and 1105 (929.9–1313) μg/ml, respectively. Ascorbic acid (standard) had an IC_50_ value of 230.7 (202.3–263.2) μg/ml. *A. nilotica* leaves, pods and bark extracts showed similar inhibition of lipid peroxidation as previously reported by Sadiq et al. [14] for DPPH (1, 1 diphenyl-2-picryl hydrazyl) inhibition.

**Table 1:** Estimated IC_50_ values for inhibition of lipid peroxidation by *Acacia nilotica* (leaves, pods and bark) and ascorbic acid (standard)

Parts of *Acacia nilotica*	IC_50_ (μg/ml)	Hill-slope
Leaves	375.5 (326.6–431.8)	1.19 (0.98–1.41)
Pods	486.5 (431.5–548.6)	1.37 (1.14–1.60)
Bark	1105 (929.9–1313)	1.10 (0.86–1.340)
Vitamin C (Control)	230.7 (202.3–263.2)	0.99 (0.84–1.142)

Parameters are presented as mean estimates (95% confidence intervals)

The lipid peroxidation induced by Fe^+2^ is a reliable system for evaluation of antioxidant potential of different plant extracts. Malondialdehyde (degradation product of lipid peroxidation) causes cell damage and form a pink chromogen with thiobarbituric acid. Kalaivani and Mathew [18], reported that ethanol fractions of *A. nilotica* leaves extract showed the highest inhibition of lipid peroxidation compared to other solvent fractions, resulting in IC_50_ values comparable to a positive control (catechin). The current study showed that ethanol extracts of *A. nilotica* leaves showed inhibition of lipid peroxidation comparable to the positive control (Vitamin C).

#### Ferric reducing antioxidant power capacity of plant extracts

**Fig. 3:**
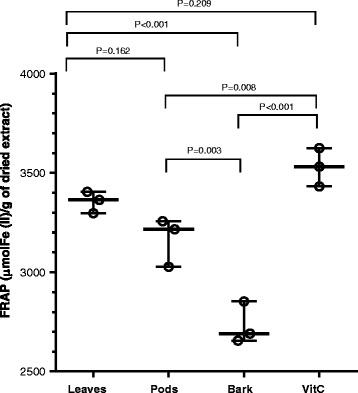
Ferric reducing antioxidant potential (FRAP) of *A. nilotica* extracts

FRAP method is based on reduction of colorless complex (Fe^+3^ -TPTZ) to blue colored complex (Fe^+2^ –TPTZ) by donation of electrons from antioxidants that results in stopping the free radical production chain. FRAP is a simple assay that gives fast and reproducible results [19]. The leaves extract showed FRAP value 3355 μM of Fe (II)/g of dried extract that was higher than the other extracts (Fig. 3). The pods and bark were found to have FRAP values 3167 μM of Fe (II)/g and 2733 μM of Fe (II)/g of dried extracts, respectively. The positive control vitamin C was found to have FRAP value of 3529 μM of Fe (II)/g that was not significantly different from leaves extract (*p* < 0.05). The ferric reducing potential of *A. nilotica* leaves, pods and bark extracts was found to be higher than that previously reported for leaves, pods and seeds of *A. lecuophloea* Roxb [20].

The phenolic compounds present in plant extracts are responsible for various biological activities like strong antioxidants, antidiabetic, antiaging, anticancer and prevention of cardiac diseases. The qualitative analysis of leaves, pods and bark extracts of *A. nilotica* by liquid chromatography-electrospray ionization-ion trap-mass spectrometer (LC-ESI-IT-MS) indicated that all the tested extracts contained phenolic compounds, belonging to the classes of gallic acid, catechin and gallocatechin derivatives [14]. The quantification of phenolic compounds, carried out by high performance liquid chromatography/diode array detection (HPLC/DAD) indicated that the leaves were rich in gallic acid (87,502 ± 151.1 mg/kg), catechin (82,588 ± 171.3 mg/kg), isoquercetin (9725 ± 41.6 mg/kg), rutin (6856 ± 15.4 mg/kg) and quercetin (1637 ± 11.6 mg/kg), whereas, apigenin (103 ± 1.7 mg/kg) and kaempferol (114 ± 2.1 mg/kg) were present in minute quantities [14]. The pods were rich in gallic acid (139,458 ± 191.9 mg/kg), tannic acid (6874 ± 31.9 mg/kg), catechin (6369 ± 29.2 mg/kg) and rutin (4026 ± 17.8 mg/kg), whereas quercetin (592 ± 5.7 mg/kg) and isoquercetin (824 ± 3.8 mg/kg) were found in minute quantities. The bark extract contained catechin (18,501 ± 71.1 mg/kg), isoquercetin (7479 ± 119.5 mg/kg), tannic acid (1459 ± 11.4 mg/kg) and quercetin (1069 ± 17.3 mg/kg) in considerable quantities.

### Antimalarial activities of *A. nilotica* extracts

**Fig. 4:**
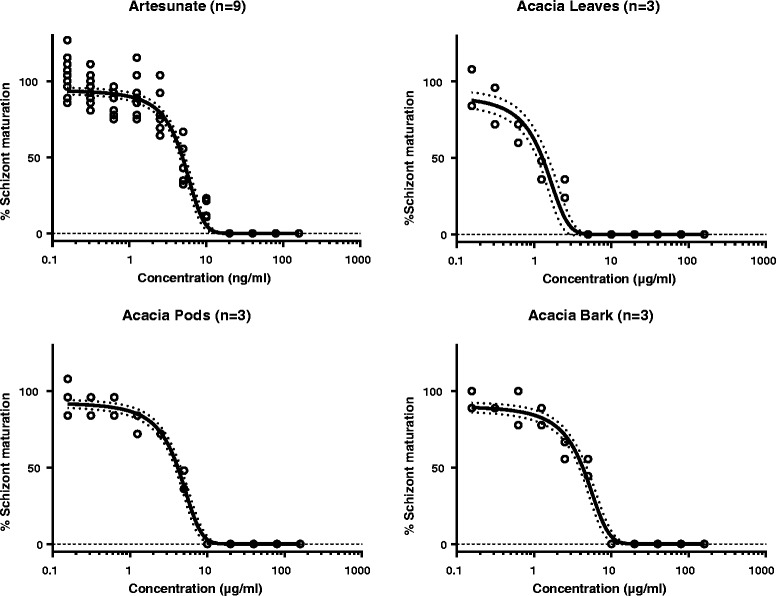
Maturation of P*. falciparum* (3D7) after 48 h incubation with *A. nilotica* extracts. Open circles represent observations, the solid lines represent the estimated mean curves, and the broken lines represent the 95% confidence intervals of the mean estimates

**Fig. 5:**
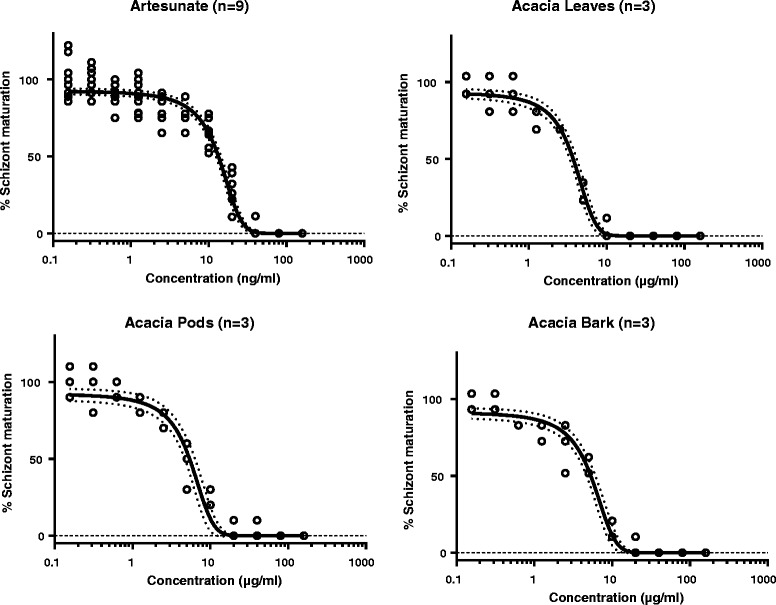
Maturation of *P. falciparum* (3D7) after 96 h incubation with *A. nilotica* extracts. Open circles represent observations, the solid lines represent the estimated mean curves, and the broken lines represent the 95% confidence intervals of the mean estimates

The antimalarial activity of *A. nilotica* extracts (leaves, pods and bark) were determined by the schizont maturation inhibition assay. Ethanol extracts of leaves, pods and bark were screened for antimalarial activity against an artesunate sensitive strain of *P. falciparum* (3D7). The schizonticide activities were expressed in terms of IC_50_ values, defined as minimum concentration of plant extracts required to inhibit 50% of schizont maturation. The schizont maturation inhibition results at 48 h and 96 h by artesunate, leaves, pods and bark extracts are presented in Figs. 4 and 5, respectively. After 48 h of incubation, leaves were found more potent than pods and bark against the plasmodium parasite with an IC_50_ of 1.29 (1.08–1.49) μg/ml (Table 2). Whereas, the pods and bark extracts resulted in IC_50_ values of 4.16 (3.79–4.53) and 4.28 (3.79–4.77) μg/ml, respectively. The 5 μg/ml of leaves showed full inhibition of mature schizonts development while pods and bark extracts showed full inhibition at 10 μg/ml. Furthermore, the parasites incubated for 96 h (after removing the drug/extract residues after 48 h) were evaluated for the development of mature schizonts. The IC_50_ values estimated for leaves, pods and bark extracts against *P. falciparum* after 96 h of incubation were 3.72 (3.34–4.11) μg/ml, 5.41 (4.59–6.22) μg/ml and 5.32 (4.63–6.01) μg/ml, respectively. The development of parasites to mature schizonts was interrupted by all extracts, suggesting schizonticidal activity of *A. nilotica* plant extracts against *P. falciparum*. *A. nilotica* is a plant rich in phenolic compounds and alkaloids that might be responsible for the antimalarial effects. *A. nilotica* root extracts showed a significant dose-dependent reduction in parasite densities of *Plasmodium berghei* in infected mice [21]. Methanol extracts of *A. nilotica* stem bark showed in vitro antimalarial effects with IC_50_ values of 73.59 ± 2.87 and 70.33 ± 1.89 μg/ml against chloroquine resistant and sensitive *P. falciparum* strains, respectively [22]. The bioactive potential of *A. nilotica* plant might be due to high total phenolic and flavonoid contents. As previously reported, total phenolic contents in leaves, pods and bark were 136.49 ± 2.49, 103.68 ± 1.46 and 62.03 ± 1.69 mg GAE/g of extract and total flavonoid contents were 37.53 ± 0.82, 29.03 ± 0.92 and 45.5 ± 2.99 mg QE/g, respectively [14].

**Table 2:** Estimated IC_50_ values for the antimalarial effects of *Acacia nilotica* (leaves, pods and bark) and artesunate (standard)

Parts of *Acacia nilotica*	IC_50_ after 48 h	IC_50_ after 96 h
Leaves (μg/ml)	1.29 (1.08–1.49)	3.72 (3.34–4.11)
Pods (μg/ml)	4.16 (3.79–4.53)	5.41 (4.59–6.22)
Bark (μg/ml)	4.28 (3.79–4.77)	5.32 (4.63–6.01)
Artesunate (ng/ml)	4.90 (4.50–5.29)	13.36 (12.32–14.39)

Parameters are presented as mean estimates (95% confidence intervals), after 48 or 96 h of incubation

Clarkson et al. [23] studied the antiplasmodial activity of various plant extracts, native to South Africa, against a chloroquine sensitive strain of *P. falciparum* (D10) and classified the plants extract with IC_50_ values ≤10 μg/ml as promising antiplasmodial activity and plants with IC_50_ values ≤5 μg/ml were considered to be highly active against the parasite. In the current study, the IC_50_ values of leaves, pods and bark extracts were all estimated to be less than 5 μg/ml (1.29, 4.16 and 4.28 μg/ml respectively), indicating a high antimalarial potential of *A. nilotica*. Moreover, Clarkson et al. [23] reported the IC_50_ values of aqueous and dichloromethane/methanol (1:1) extracts of *A. nilotica* twigs against *P. falciparum* (D10) as 32 and 13 μg/ml, respectively, while the parasites (*P. falciparum,* 3D7*)* tested in the current study were found more susceptible (i.e. lower IC_50_ values) to leaves, pods and bark extracts of *A. nilotica*. In a previous research report methanol extracts of *A. nilotica* seed and husk showed IC_50_ values less than 5 μg/ml against both chloroquine sensitive and resistant strains of *P. falciparum* [24]. The reported variation might be due to different plasmodium strains, extraction solvents and/or due to variations in the phytoconsituents content present in different parts of the plant. The phytoconsituents content are also known to be affected by plant species, maturity, growing conditions, soil conditions and post-harvest treatment [25]. The leaves of the *A. nilotica* were found to be rich in phenolic compounds compared to pods and bark extracts [14]. This might explain the higher antimalarial activity of leaves compared to pods and bark. The phenolic compounds in plant extracts have been shown previously to exhibit antimalarial effects [26], supporting the higher efficacy of *A. nilotica* leaves due to high phenolic content. The IC_50_ value of *Artemisia annua* crude extract against *P. falciparum* was reported to be 3.9 μg/ml [27]. The leaves of *A. nilotica* showed IC_50_ values (1.29 μg/ml) lower than the crude extract of *Artemisia annua*, indicating *A. nilotica* as an important potential source of new antimalarial compounds.

## Conclusion

The leaves, pods and bark extracts of *A. nilotica* showed potent antimalarial and antioxidant activities. The results highlighted that the plant can be used as a source of natural antioxidants and antiplasmodial compound. The remarkable antimalarial activity of *A. nilotica* encourages the investigation of native and naturalized Asian plants to explore as a potential source of antimalarial drugs. Further purification, isolation and identification of active compounds are needed to develop novel antimalarial drugs and to characterize in vivo effects and antimalarial mechanism of action.

## Abbreviations

FRAP: Ferric reducing antioxidant power
PBS: Phosphate buffer solution
TBARS: Thiobarbituric acid reactive substances
TCA: Trichloro acetic acid
TPTZ: 2, 4, 6-tripyridyl-s-triazine

## References

[1] Ambasta SP (1994). The useful plants of India, publication and information directorate.

[2] Maldini M, Montoro P, Hamed AI, Mahalel UA, Oleszek W, Stochmal A, Piacente S (2011). Strong antioxidant phenolics from *Acacia nilotica*: profiling by ESI-MS and qualitative–quantitative determination by LC–ESI-MS. J Pharm Biomed Anal.

[3] Hussein G, Miyashiro H, Nakamura N, Hattori M, Kakiuchi N, Shimotohno K (2000). Inhibitory effects of Sudanese medicinal plant extracts on hepatitis C virus (HCV) protease. Phytother Res.

[4] Sadiq MB, Tarning J, Aye Cho TZ, Anal AK (2017). Antibacterial activities and possible modes of action of *Acacia nilotica* (L.) Del. Against multidrug-resistant *Escherichia coli* and salmonella. Molecules.

[5] Singh R, Singh B, Singh S, Kumar N, Kumar S, Arora S (2008). Anti-free radical activities of kaempferol isolated from *Acacia nilotica* (L.) Willd. Ex. del. Toxicol in Vitro.

[6] Tamuly C, Hazarika M, Bora J, Bordoloi M, Boruah MP, Gajurel P (2015). In vitro study on antioxidant activity and phenolic content of three piper species from north East India. J Food Sci Technol.

[7] Tuncel NB, Yılmaz N (2015). Optimizing the extraction of phenolics and antioxidants from feijoa (*Feijoa sellowiana*, Myrtaceae). J Food Sci Technol.

[8] World Health Organization (2015). World malaria report 2015.

[9] Cui L, Mharakurwa S, Ndiaye D, Rathod PK, Rosenthal PJ (2015). Antimalarial drug resistance: literature review and activities and findings of the ICEMR network. Am J Trop Med Hyg.

[10] Anthony MP, Burrows JN, Duparc S, JMoehrle J, Wells TN (2012). The global pipeline of new medicines for the control and elimination of malaria. Malar J.

[11] Adwan, G., Abu-Shanab, B., & Adwan, K. (2010). Antibacterial activities of some plant extracts alone and in combination with different antimicrobials against multidrug-resistant *Pseudomonas aeruginosa* strains. Asian Pac J Trop Med, 3(4), 266-266.

[12] Nabavi SM, Ebrahimzadeh MA, Nabavi SF, Hamidinia A, Bekhradnia AR (2008). Determination of antioxidant activity: phenol and flavonoids content of *Parrotia persica Mey*. Pharmacol Online.

[13] Ohkawa H, Ohishi N, Yagi K (1979). Assay for lipid peroxides in animal tissues by thiobarbituric acid reaction. Anal Biochem.

[14] Sadiq MB, Hanpithakpong W, Tarning J, Anal AK (2015). Screening of phytochemicals and in vitro evaluation of antibacterial and antioxidant activities of leaves, pods and bark extracts of *Acacia nilotica* (L.) del. Ind Crop Prod.

[15] Wong CC, Li HB, Cheng KW, Chen F (2006). A systematic survey of antioxidant activity of 30 Chinese medicinal plants using the ferric reducing antioxidant power assay. Food Chem.

[16] Chotivanich K, Tripura R, Das D, Yi P, Day NP, Pukrittayakamee S, White NJ (2014). Laboratory detection of artemisinin-resistant *Plasmodium falciparum*. Antimicrob Agents Chemother.

[17] Gordon MH, Hudson BJF (1990). The mechanism of the antioxidant action in vitro. Food antioxidants.

[18] Kalaivani T, Mathew L (2010). Free radical scavenging activity from leaves of *Acacia nilotica* (L.) wild. Ex Delile, an Indian medicinal tree. Food Chem Toxicol.

[19] Benzie IF, Strain JJ (1996). The ferric reducing ability of plasma (FRAP) as a measure of “antioxidant power”: the FRAP assay. Anal Biochem.

[20] Zia-Ul-Haq M, Ćavar S, Qayum M, Khan I, Ahmad S (2013). Chemical composition and antioxidant potential of *Acacia leucophloea* Roxb. Acta Botanica Croatica.

[21] Alli LA, Adesokan AA, Salawu AO (2016). Antimalarial activity of fractions of aqueous extract of *Acacia nilotica* root. J Intercultural Ethnopharmacol.

[22] Kirira PG, Rukunga GM, Wanyonyi AW, Muregi FM, Gathirwa JW, Muthaura CN, Ndiege IO (2006). Anti-plasmodial activity and toxicity of extracts of plants used in traditional malaria therapy in Meru and Kilifi districts of Kenya. J Ethnopharmacol.

[23] Clarkson C, Maharaj VJ, Crouch NR, Grace OM, Pillay P, Matsabisa MG, Folb PI (2004). In vitro antiplasmodial activity of medicinal plants native to or naturalised in South Africa. J Ethnopharmacol.

[24] El-Tahir A, Satti GM, Khalid SA (1999). Antiplasmodial activity of selected Sudanese medicinal plants with emphasis on *Acacia nilotica*. Phytother Res.

[25] Jeffery EH, Brown AF, Kurilich AC, Keck AS, Matusheski N, Klein BP, Juvik JA (2003). Variation in content of bioactive components in broccoli. J Food Compos Anal.

[26] Ntie-Kang F, Onguéné PA, Lifongo LL, Ndom JC, Sippl W, Mbaze LMA (2014). The potential of anti-malarial compounds derived from African medicinal plants, part II: a pharmacological evaluation of non-alkaloids and non-terpenoids. Malar J.

[27] O'neill MJ, Bray DH, Boardman P, Phillipson JD, Warhurst DC (1985). Plants as sources of antimalarial drugs part. 1. In vitro test method for the evaluation of crude extracts from plants. Planta Med.

